# Molecular docking analysis of glycogen phosphorylase with inhibitors from *Cissampelos pareira* Linn.

**DOI:** 10.6026/97320630017866

**Published:** 2021-10-31

**Authors:** Kirubhanand Chandrashekar, Ponnulakshmi Rajagopal, Shazia Fathima JH, Saravanan Radhakrishnan, Vijaya Prakash Krishnan Muthaiah, Bharat Ramrao Sontakke, Vishwajit Ravindra Deshmukh, Vijayalakshmi Periyasamy, Gayatri Girish Muthiyan, Aaditya Madhusudan Tarnekar, TS Gugapriya, Patil Ashlesh Laxman, Satyendra Chandra Tripathi, Selvaraj Jayaraman

**Affiliations:** 1 Department of Anatomy, AIIMS, Nagpur 441108, India; 2 Central Research Laboratory, Meenakshi Academy of Higher Education and Research (Deemed to be University), Chennai-600 078, India; 3 Department of Oral and Maxillofacial Pathology, Ragas Dental College and Hospitals, Chennai, India; 4 Department of Biochemistry, Karapaga Vinayaga Institute of Medical and Dental science, Madhuranthagam- 603308, Tamil Nadu, India; 5 Department of Rehabilitation Science, School of Public Health and Health professions, 633 Kimball, Buffalo NY, 14214-3079; 6 DBT-BIF Centre, PG & Research Department of Biotechnology & Bioinformatics, Holy Cross College (Autonomous), Trichy, Tamilnadu, India; 7 Department of Physiology, AIIMS, Nagpur 441108, India; 8 Department of Biochemistry, AIIMS, Nagpur 441108, India; 9 Department of Biochemistry, Saveetha Dental College and Hospitals, Saveetha Institute of Medical and Technical Sciences, Saveetha University, Chennai - 600 077, India

**Keywords:** Diabetes mellitus, *Cissampelos pareira*, Glycogen phosphorylase, Molecular docking

## Abstract

*Cissampelos pareira* Linn. is a climbing herb known in Indian traditional medicine as laghupatha. It belongs to the Menispermaceae family. The enzyme glycogen phosphorylase (GP) is a promising target for the treatment of type-2 diabetes (T2DM). A variety of natural product inhibitors with both pharmaceutical and nutraceutical potential have been reported in the search for powerful, selective and drug-like GP inhibitors that could lead to hypoglycemic medicines. Therefore, it is of interest to document the molecular docking analysis data of glycogen phosphorylase with compounds from *Cissampelos pareira* Linn. We report the optimal binding features of 4 compounds namely Trans-N-feruloyltyramine, Coclaurine, Magnoflorine, and Curine with the target protein for further consideration in the context of T2DM.

## Background:

Diabetes is a metabolic condition that affects hundreds of millions of people around the world. Type-2 diabetes mellitus (T2DM) is characterized by decreased insulin secretion by the Islets of Langerhans cells and insulin resistance [1]. This leads to hyperglycemia, delayed or impaired wound healing, diabetic retinopathy, diabetic nephropathy, diabetic neuropathy, and other complications [2]. Glycogen phosphorylase (GP) is a major enzyme in the glycogenolysis pathway that catalyzes glycogen breakdown to glucose-1-phosphate (Glc-1-P). It is a desirable target for the development of hypoglycemic drugs [3]. The efficacy of GP inhibitors on blood glucose control and hepatic glycogen balance is known [4]. Natural products are the source of the bulk of today's therapeutic medications [5-6]. In essence, natural product exploitation in drug discovery initiatives can guide the development of new medications [6]. Evolutionary selection has resulted in natural compounds with exceptional potency and selectivity. After identifying a natural product as a potential lead candidate for further development, structural changes utilizing organic synthesis methods enable the production of more selective and efficient therapeutic agents for a given target. Furthermore, using the GP as a target, the identification of safe and efficacious natural compounds with the ability to help regulate blood glucose levels could lead to nonprescription nutraceutical (or functional food) options [7]. *Cissampelos pareira*, a well-known medicinal climber-plant belonging to the Menispermaceae family, has long been utilized in traditional medicine. Since ancient times, it has been used to cure a variety of ailments including ulcers, wounds, rheumatism, fever, asthma, cholera, diarrhea, inflammation, snakebite, malaria, rabies, and blood purification. Non-healing ulcers, skin problems, scabies, leprosy, migraine, leucorrhoea, and gonorrhea are also treated with it. Therefore, it is of interest to document the molecular docking analysis data of glycogen phosphorylase with compounds from *Cissampelos pareira* Linn.

## Materials & Methods:

### Protein Preparation:

The 3D structures of Glycogen phosphorylase were obtained from Protein Data Bank (Pdb id: 1L5Q) in the pdb format. Protein macromolecules were separated from solvents and nonstandard ligands or residues using Autodock tools and were saved in the .pdb format. Macromolecules were optimized by adding hydrogen atoms tools and were saved in the pdbqt format.

### Ligand Preparation

Eleven bioactive compounds (Table 1 -see PDF) from *Cissampelos pareira* were selected from literature and the structure of each compound was downloaded from PubChem database as .sdf format. The SDF format of the selected ligands was converted into their 3D PDB formats using online smile translator and loaded to PyR. The geometry of each compounds were optimized with PyRx in the pdbqt format.

### Molecular Docking studies:

Binding mode and interaction of Glycogen phosphorylase with individual chemical constituents of *Cissampelos pareira* were performed PyRx [8-10]. Docking was performed to obtain a population of possible conformations and orientations for the ligand at the binding site. The protein was loaded in PyRx software, creating a PDBQT file that contains a protein structure with hydrogens in all polar residues. All bonds of ligands were set to be rotatable. The docking site on protein target was defined by establishing a grid box with the dimensions of X: 54.5255, Y: -60.4211, Z: 100.1374 and number of points on X: 25, Y: 25 and Z: 25. The best conformation was chosen with the lowest docked energy, after the docking search was completed. The ligand-protein complexes were visualized with PyMol to see the interactions model between compounds and target amino acid protein residues.

## Results and Discussion:

Molecular docking is a technique for predicting the binding orientation of small molecules and drug candidates to their protein targets, as well as their affinity and activity [9,10]. AutoDock Vina in PyRx 0.8 was used to perform the molecular docking analysis. AutoDock Vina is a molecular docking and virtual screening program that can operate on multiple cores and threads. The value of binding free energy (G binding) was used to represent the affinity between ligands and receptors. The sum of total intermolecular energy, total internal energy, and torsional free energy minus the energy of the unbound system was used to determine binding free energy. The optimum interaction pose was determined by the conformational with the lowest energy binding value. We chose the best four compounds based on binding energy. The energy binding values of chemical components in *Cissampelos pareira* were shown in Table 2(see PDF). The best affinities to the target protein Glycogen phosphorylase were Trans-N-feruloyltyramine, Coclaurine, Magnoflorine, and Curine.

The interaction between the ligand and the target protein was investigated using Pymol. The interaction of Trans-N-feruloyltyramine in the binding pocket of Glycogen phosphorylase was shown in Figure 1a(see PDF) (green color represents the compound). This interaction visualization was utilized to see how well the ligand conformed to the target protein and to identify the amino acid residues that were involved in the interaction (Table 2 - see PDF). Trans-N-feruloyltyramine interacted with 5 amino acid residues of Glycogen phosphorylase ASN-133, LEU-136, TYR-280, ASN-282 and ASN-284. More number of hydrogen bond interactions with target protein indicates that compounds have good binding affinity. Hydrogen bond distance of each bonds are 1.8, 1, 3, 2.3, 2.4 and 2.6Å respectively. Figure 1b(see PDF) represents the interaction between the Coclaurine and Glycogen phosphorylase. Coclaurine showed strong binding with Glycogen phosphorylase with binding energy of -8.3kcal/mol. And also its formed the three hydrogen bonds interaction with the residues ASP-283, HIS-377, LYS-574 and distance of hydrogen bonds is 1.9,2.5 and 2.9 respectively. Figure 1c(see PDF) exemplifies the docking conformation of Glycogen phosphorylase with Magnoflorine. The interactions of hydrogen bonds between Glycogen phosphorylase and Magnoflorine were visualized as Figure 1c(see PDF). Table 2(see PDF) represents the docking energy and the bonding distance between Glycogen phosphorylase with Magnoflorine. This study shows that this complex is stabilized by the hydrogen bonds of bond length 1.9 Å and 2.7 Å with the residues ASN-283 and LYS-574 of Magnoflorine, respectively. Hydrogen bonds have been identified as important interactions in determining the binding energy and stability of these receptor-ligand complexes. The binding energy of the lowest energy conformer of Glycogen phosphorylase with Magnoflorine complex was calculated computationally and found to be -7.6 Kcal/mol.

Figure 1d(see PDF) illustrates the docking conformation of Curine with Glycogen phosphorylase. Table 2(see PDF) shows the docking energy and the bonding distance between Curine with Glycogen phosphorylase. The amino acids interacts with Curine are found to be ASN-284, LYS-574 and LYS-568. The docking complex is stabilized by the hydrogen bonds of distance was 1.8, 2.5 and 1.5 Å. The docking energy for Curine with Glycogen phosphorylase was found to be –5.5 Kcal/mol. The binding affinity value is affected by the type and quantity of bonds established between the ligand and the receptor. Because hydrogen bonds improve the contact effect between proteins and ligands, they affect protein stability [11]. The binding affinity value rises with the number of hydrogen bonds. Glycogen phosphorylase (GP) catalyzes glycogen breakdown to produce glucose-1-phosphate. The 280's loop (residues 280–288), which lies between -strand 11 and -helix 8, is expected to play a vital function in substrate and inhibitor binding regulation [11,12]. We show four ligands have active site affinity. Trans-N-feruloyltyramine had the strongest affinity. Glycogen phosphorylase, which is responsible for the production of glucose, is one of the reasons of hyperglycemia. Different ligands found in *Cissampelos pareira* may inhibit this enzyme, resulting in a significant improvement in diabetics' glycemic state. The most polyvalent molecule with the best affinities to selected receptor is clearly Trans-N-feruloyltyramine, followed by Coclaurine, Magnoflorine, and Curine, which had the lowest binding affinities among the examined series.

## Conclusion:

We report the optimal binding features of 4 compounds namely Trans-N-feruloyltyramine, Coclaurine, Magnoflorine, and Curine with the target protein GP for further consideration in the context of T2DM.
